# *Argonaute2* Mediates Compensatory Expansion of the Pancreatic β Cell

**DOI:** 10.1016/j.cmet.2013.11.015

**Published:** 2014-01-07

**Authors:** Sudhir G. Tattikota, Thomas Rathjen, Sarah J. McAnulty, Hans-Hermann Wessels, Ildem Akerman, Martijn van de Bunt, Jean Hausser, Jonathan L.S. Esguerra, Anne Musahl, Amit K. Pandey, Xintian You, Wei Chen, Pedro L. Herrera, Paul R. Johnson, Donal O’Carroll, Lena Eliasson, Mihaela Zavolan, Anna L. Gloyn, Jorge Ferrer, Ruby Shalom-Feuerstein, Daniel Aberdam, Matthew N. Poy

**Affiliations:** 1 Max Delbrueck Center for Molecular Medicine, 13125 Berlin, Germany; 2 Institut National de la Santé et de la Recherche Médicale (INSERM), UMR-S 976, 75475 Paris, France; 3 Université Paris Diderot, Skin Research Center, 75013 Paris, France; 4 Department of Anatomy and Cell Biology, The Ruth and Bruce Rappaport Faculty of Medicine, The Technion-Israel Institute of Technology, 31096 Haifa, Israel; 5 Genomic Programming of Beta-Cells Laboratory, Institut d’Investigacions Biomèdiques August Pi I Sunyer (IDIBAPS), 08036 Barcelona, Spain; 6 Department of Medicine, Imperial College London, W12 0NN London, UK; 7 Oxford Centre for Diabetes, Endocrinology, & Metabolism, University of Oxford, OX3 7LE Oxford, UK; 8 Computational and Systems Biology, Biozentrum, University of Basel, 4056 Basel, Switzerland; 9 Department of Clinical Sciences, Lund University Diabetes Center, Lund University, Malmö University Hospital, SE-205 02 Malmö, Sweden; 10 Department of Genetic Medicine and Development, Faculty of Medicine, University of Geneva, 1211 Geneva, Switzerland; 11 NIHR Oxford Biomedical Research Centre, ORH Trust, OCDEM, Churchill Hospital, OX3 7LJ Oxford, UK; 12 Nuffield Department of Surgery, University of Oxford, OX3 9DU Oxford, UK; 13 European Molecular Biology Laboratory, 00015 Monterotondo Scalo, Italy

## Abstract

Pancreatic β cells adapt to compensate for increased metabolic demand during insulin resistance. Although the microRNA pathway has an essential role in β cell proliferation, the extent of its contribution is unclear. Here, we report that *miR-184* is silenced in the pancreatic islets of insulin-resistant mouse models and type 2 diabetic human subjects. Reduction of *miR-184* promotes the expression of its target *Argonaute2* (*Ago2*), a component of the microRNA-induced silencing complex. Moreover, restoration of *miR-184* in *leptin*-deficient *ob/ob* mice decreased *Ago2* and prevented compensatory β cell expansion. Loss of *Ago2* during insulin resistance blocked β cell growth and relieved the regulation of *miR-375*-targeted genes, including the growth suppressor *Cadm1*. Lastly, administration of a ketogenic diet to *ob/ob* mice rescued insulin sensitivity and *miR-184* expression and restored *Ago2* and β cell mass. This study identifies the targeting of *Ago2* by *miR-184* as an essential component of the compensatory response to regulate proliferation according to insulin sensitivity.

## Highlights

• Silencing of *miR-184* during insulin resistance promotes its target Ago2
• Loss of *Ago2* during insulin resistance blocks pancreatic β cell proliferation
• *Ago2* mediates the suppression of *Cadm1* by *miR-375* in the β cell
• Administration of the ketogenic diet to *ob/ob* mice rescues *miR-184* in islets

## Graphical Abstract

**Figure:**
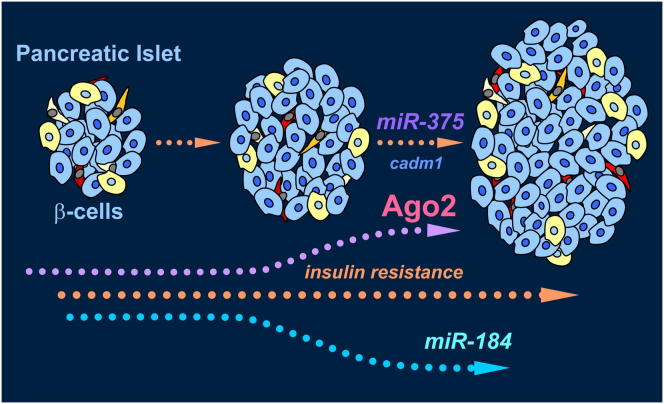

## Teaser

Tattikota et al. find that as β cells adapt to increased metabolic demand during insulin resistance in obesity, miR-184 is silenced to alleviate repression of its target Argonaute2, a component of the microRNA-induced silencing complex. Argonaute2 promotes compensatory β cell proliferation via miR-375 and its target genes, including the growth suppressor Cadm1.

## Introduction

Adaptation to environmental stress is a fundamental cellular process that promotes the maintenance of the physiologic steady state (Spriggs et al., 2010). Stress responses have been shown to induce numerous changes, such as activation of gene expression programs, which have evolved to allow for the cell to promote its own survival (Kültz, 2005, Ebert and Sharp, 2012). For example, in response to insulin resistance, the pancreatic β cell undertakes measures to proliferate and increase its output of secreted insulin. A coordinated increase in both β cell mass and secretory function constitutes the compensatory response to maintain normoglycemia (Muoio and Newgard, 2008). Although the underlying mechanisms directing these processes are still not completely understood, several studies have illustrated a role for metabolic changes in catalyzing β cell expansion (Steil et al., 2001). Furthermore, cellular pathways enabling the β cells to proliferate and adapt to increases in metabolic load may act by ultimately promoting signaling cascades essential to increasing both secretion and islet mass (Rhodes, 2005).

Recent evidence has shown the microRNA (miRNA) pathway as an important regulator of gene expression in response to metabolic stress (Leung and Sharp, 2010). Central to this mechanism are the Argonaute (Ago) proteins, which mediate this pathway by facilitating the interaction between miRNAs and their target mRNAs (Höck and Meister, 2008, Bartel, 2009). In addition, Ago proteins have been shown to accumulate in stress granules upon exposure to oxidative stress; however, their role in this compartment is not understood (Leung et al., 2006). Although loss of Argonaute2 (Ago2) expression in the MIN6 β cell line model resulted in enhanced secretion, its role in the stress response of the β cell has not been described (Tattikota et al., 2013).

We have previously shown that loss of *miR-375* expression, among the most abundant miRNA in the pancreatic islet, inhibited the compensatory β cell proliferation in *leptin*-deficient *ob/ob* mice and resulted in severe hyperglycemia and diabetes (Poy et al., 2009). The absence of any dramatic effect on the development or specification of the different cell populations in the *miR-375* knockout mouse may indicate a larger role for this miRNA in stress responses (Mendell and Olson, 2012). Furthermore, these observations suggest that many of the targets of *miR-375* are also relevant to the adaptive response of the β cell and likely play a role in proliferation during metabolic stress. Although extensive sequencing efforts have identified ∼2,000 mature miRNA sequences in human tissues, relatively little is understood regarding how small RNAs coordinately function in these cellular processes (Kozomara and Griffiths-Jones, 2011).

Here, we show that *miR-184* is silenced during insulin resistance to promote the expression of Ago2 in the pancreatic β cell. Deletion of *Ago2* in *ob/ob* mice reduced compensatory proliferation of this cell type, thereby underlining an integral role for the miRNA pathway in this process. Moreover, we observed that Ago2 mediates the function of *miR-375* in regulating the growth suppressor *Cadm1*. Taken together, our results show that several components of the miRNA pathway contribute in a concerted effort to facilitate proliferation of the β cell to meet metabolic demand during insulin resistance.

## Results

### Silencing of *miR-184* in the Pancreatic β-Cell Promotes Its Target Argonaute2

**Figure 1:**
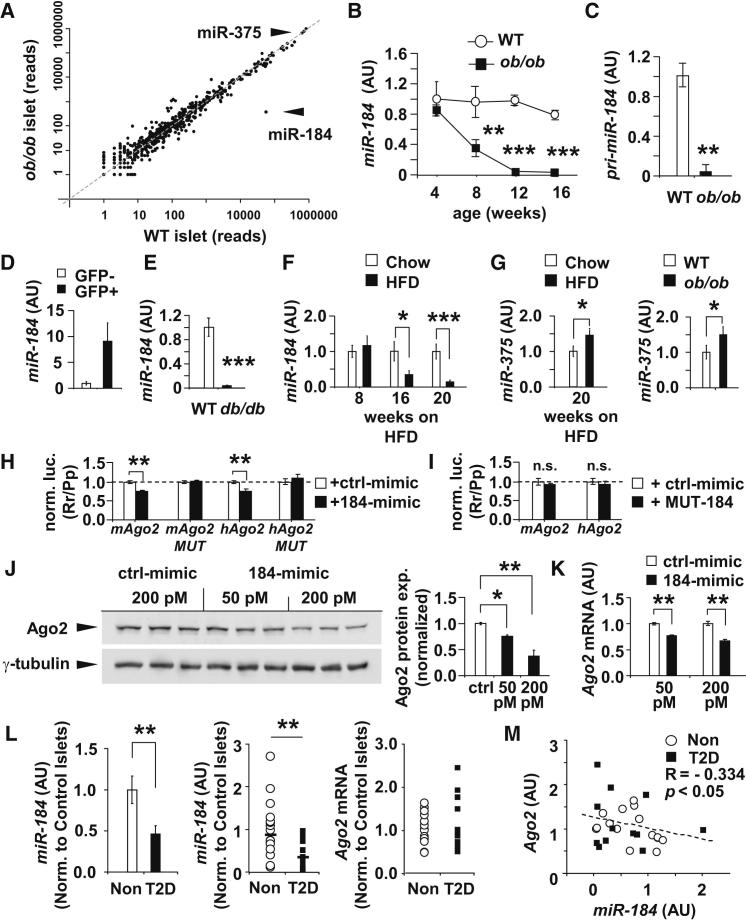
*miR-184* Is Silenced during Insulin Resistance and Directly Targets *Argonaute2* (A) Comparison of small RNA sequencing analysis from total RNA from islets of 12-week-old *ob/ob* and wild-type (WT) littermates. (B) Quantitative real-time PCR analysis of *miR-184* in islets of *ob/ob* and WT mice from 4–16 weeks of age (n = 3–5). (C) Quantitative real-time PCR analysis of pri-*miR-184* in islets of *ob/ob* mice and WT littermates at 16 weeks of age (n = 3–5). (D) Quantitative real-time PCR analysis of *miR-184* in FACS-sorted β cells from 16-week-old mouse *insulin* promoter-GFP mice (n = 6). (E) Quantitative real-time PCR analysis of *miR-184* in islets of *db/db* mice and WT littermates at age 12 weeks (n = 4). (F) Quantitative real-time PCR analysis of *miR-184* in islets of C57BL/6 mice on high-fat diet (HFD) or chow diet. (G) Quantitative real-time PCR analysis of *miR-375* in islets of C57BL/6 mice on HFD or chow diet and in islets of *ob/ob* mice and littermates at age 12 weeks (n = 6). (H and I) Luciferase assays in MIN6 cells testing direct targeting of mouse and human *Ago2* genes by *miR-184* (184-mimic) or mutant (MUT-184). (J and K) Western blot and quantitative real-time PCR analysis of *Ago2* after transfection of *miR-184*-mimic and scrambled control. (L) Quantitative real-time PCR analysis of *miR-184* and *Ago2* in islets from nondiabetic (Non) and type-2 diabetic human subjects (T2D) after normalization to *RNU6b* and *TBP*, respectively, expressed as fraction of control islets. p values represent a Mann-Whitney significance test (p = 0.009 for *miR-184*). (M) Correlation of quantitative real-time PCR analysis from (L) of *Ago2* and *miR-184* in individual T2D (n = 12) and nondiabetic (n = 15) human subjects. Results presented as mean ± SEM. ^∗^p < 0.05; ^∗∗^p < 0.01; ^∗∗∗^p < 0.001. See also Figure S1 and Tables S1 and S2.

In light of the essential role of *miR-375* in adaptive growth of the pancreatic β cell, we first sought to identify the additional components of the miRNA pathway that coordinately mediate this mechanism. We performed small RNA sequencing on total RNA from islets of 12-week-old *ob/ob* mice (Table S1 available online). Consistent with results by Zhao et al. (2009), expression of *miR-184* was the most reduced miRNA identified (Figure 1A; Table S1). We then measured *miR-184* in the islets of *ob/ob* mice from age 4–16 weeks and observed the decrease in expression starting at 8 weeks of age with the onset of resistance (Figures 1B, S1A, and S1B). Similarly, the pri-*miR-184* transcript in the islets of *ob/ob* mice by quantitative real-time PCR was also silenced, indicating that this miRNA is regulated on a transcriptional level (Figure 1C). As recently described, *miR-184* is enriched in pancreatic β cells as shown by quantitative real-time PCR from fluorescence-activated cell sorting (FACS)-sorted, GFP-positive β cells (MIP-GFP) (Figure 1D) (Hara et al., 2003, van de Bunt et al., 2013). We also observed a similar loss of expression of mature *miR-184* in the islets of 12-week-old *leptin receptor*-deficient *db/db* mice and in mice on a high-fat diet (HFD; 60% calories from fat), all of which showed that this observation is not limited to one mouse model of obesity and insulin resistance (Figures 1E and 1F). In contrast to *miR-184*, *miR-375* was modestly increased in HFD-fed animals as previously observed in *ob/ob* mice (Figure 1G) (Poy et al., 2009). In addition, the suppression of *miR-184* was not observed in the eye of *ob/ob* mice, the highest site at which expression has been measured (Figures S1C and S1D).

In order to identify direct targets of *miR-184*, we induced its expression in the MIN6 line with doxycycline after transfection with plasmids expressing the rtTA transactivator and *miR-184* under control of the operator sequence of the *Escherichia coli* tetracycline-resistance operon (*184-tetO*). Illumina WG6v2 arrays measured changes in gene expression after 16 hr of treatment with doxycycline in triplicate (Figure S1E). Several computationally predicted genes for *miR-184* were downregulated in both the array and luciferase-based experiments, including *Ago2* (Figure 1H; Figures S1F–S1H) (Friedman et al., 2009). We cloned a portion of the mouse 3′ UTR of *Ago2* (912 nt) and the complete human UTR of *Ago2* (899 nt) into a luciferase reporter construct and observed decreased activity in the presence of the *miR-184* mimic. In addition, mutating six nucleotides in the binding site within the mouse or human UTR (at position 152) from UCCGUCC to CGGGCGG abolished the inhibitory effect of *miR-184* (Figure 1H). Conversely, transfecting a mutant *miR-184* mimic had no effect on reporter activity from constructs bearing either the mouse or human *Ago2* 3′ UTR sequences (Figure 1I). Western blotting after overexpression of *miR-184* resulted in an approximately 60% decrease in Ago2 protein levels, whereas quantitative real-time PCR revealed a 40% decrease in *Ago2* mRNA in MIN6 cells (Figures 1J and 1K).

To address the relevance of this microRNA:target interaction in the islets of human subjects, we measured the expression of *miR-184* and *Ago2* in the pancreatic islets from 15 nondiabetic and 12 type 2 diabetic (T2D) donors. *miR-184* was significantly decreased in T2D islets, indicating that the expression of this specific miRNA is linked to changes in metabolic status in both mice and humans (Figure 1L; Table S2). Whereas levels of *Ago2* were not statistically significantly increased in T2D donor islets compared to the nondiabetic cohort, the inverse correlation between *Ago2* and *miR-184* expression was significant across the entire cohort (Figure 1M).

### Argonaute2 Regulates β-Cell Proliferation

**Figure 2:**
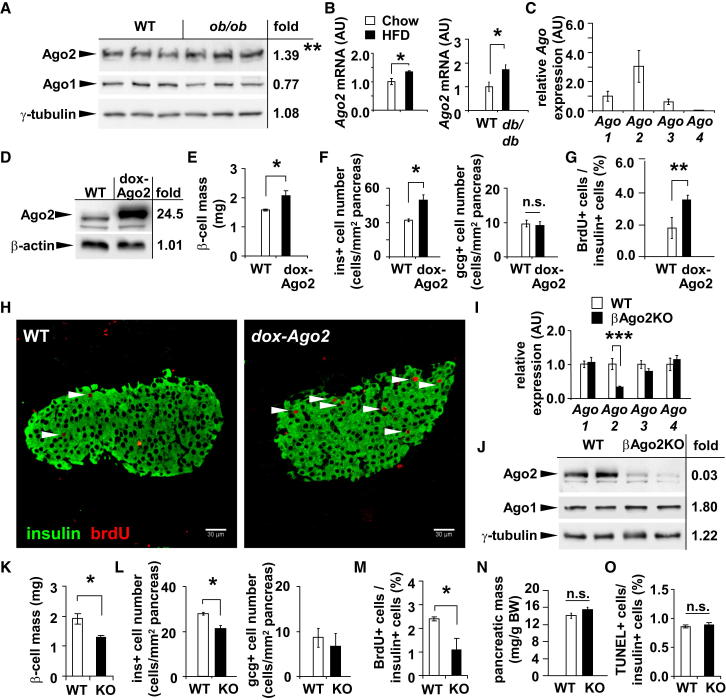
*Argonaute2* Is Increased during Insulin Resistance and Promotes β-Cell Proliferation (A) Western blot analysis of Ago1 and Ago2 from islets of 8-week-old *ob/ob* mice and wild-type (WT) littermates. (B) Quantitative real-time PCR analysis of *Ago2* in islets after 16 weeks HFD and from 12-week-old *db/db* mice (n = 3–6). (C) Quantitative real-time PCR analysis of *Argonaute* genes in FACS-sorted β cells from 12-week-old MIP-GFP mice (n = 3). (D) Western blot analysis of Ago2 from islets of 10-week-old *dox-Ago2* mice (line 30) and WT littermates. (E) β cell mass analysis of 10-week-old *dox-Ago2* mice (line 30) and WT (n = 3). (F) Morphometric analysis of insulin+ and glucagon+ cells in *dox-Ago2* and WT at age 10 weeks (n = 5–6). (G and H) Ratio of BrdU (red) and insulin+ cells (green) in 10-week-old *dox-Ago2* mice and WT littermates (n = 4) after immunostaining. Scale bars = 30 μm. (I) Quantitative real-time PCR analysis of *Argonaute* family members in islets of 10-week-old βAgo2KO mice and littermates (n = 4). (J) Western blot analysis of Ago2 and Ago1 from islets of 10-week-old βAgo2KO mice and WT. (K) β cell mass analysis of 10-week-old βAgo2KO and WT mice (n = 3). (L) Morphometric analysis of insulin+ and glucagon+ cells in βAgo2KO and littermates at 10 weeks of age (n = 5–6). (M) Ratio of BrdU+ and insulin+ cells in βAgo2KO and littermates at 10 weeks of age (n = 5–6). (N) Pancreatic mass to total body mass ratio of βAgo2KO and littermates at 10 weeks of age (n = 5). (O) Ratio of TUNEL+ and insulin+ cells in βAgo2KO and littermates (n = 3). Results presented as mean ± SEM. ^∗^p < 0.05; ^∗∗^p < 0.01. See also Figure S2.

We next measured Ago2 expression in the islets of insulin-resistant *ob/ob* mice and observed increased levels at 8 weeks of age, whereas Ago1 was not altered (Figure 2A). Similarly, Ago2 levels were increased in islets isolated from 12-week-old *db/db* mice and C57BL/6 mice on a HFD for 16 weeks (Figures 2B). As shown in other tissues, *Ago2* is the most abundant of the Ago family in GFP-positive β cells as measured by quantitative real-time PCR (Figures 2C and S2A) (Wang et al., 2012). To understand the contribution of *Ago2* to growth and function of the β cell, we generated transgenic mice bearing the mouse *Ago2* cDNA under the regulatory control of a tetracycline-responsive promoter element and crossed them with mice expressing the reverse tetracycline-controlled transactivator (rtTA) protein under the control of the rat *insulin2* (Ins-rtTA) promoter as described (*dox-Ago2*) (Nir et al., 2007). Administration of doxycycline via drinking water for 30 days to four different lines (14, 30, 61, and 69) resulted in ∼1.5- to 25-fold increase of Ago2 protein levels in isolated islets (Figures 2D and S2B). Characterization of all lines showed no changes in body weight, random glucose, or insulin levels. After a glucose challenge, *dox-Ago2* mice (line 30) exhibited transiently elevated glucose levels compared to littermates; however, no difference was observed in insulin sensitivity (Figures S2C and S2D). Moreover, acute-phase plasma insulin levels were diminished after glucose challenge, indicating reduced insulin release (Figure S2E). Morphometric analysis in *dox-Ago2* (line 30) animals showed increased β cell mass in addition to increased insulin-positive cell number and BrdU incorporation compared to littermates (Figures 2E–2H).

We then established the conditional deletion in β cells by crossing mice bearing the floxed allele of the *Ago2* gene with animals expressing Cre-recombinase under control of the rat *insulin* promoter (βAgo2KO) (O’Carroll et al., 2007, Herrera, 2000). Quantitative RT-PCR and western blotting analysis confirmed an efficient reduction in *Ago2* expression in islets from βAgo2KO mice compared to littermate controls (Figures 2I and 2J). In contrast, Ago1 was upregulated in islets isolated from βAgo2KO mice, suggesting a degree of compensation by this family member on the protein level (Figure 2J). Both random-fed and fasted glucose and insulin levels were unchanged; however, βAgo2KO mice exhibited improved glucose tolerance without any alteration in insulin sensitivity at 10 weeks of age compared to littermates (Figures S2F and S2G). As previously observed in the MIN6 model, loss of *Ago2* expression in vivo resulted in enhanced secretion of insulin after glucose challenge (Figure S2H) (Tattikota et al., 2013). In contrast to *dox-Ago2* mice, we observed decreased β cell mass and number, BrdU incorporation, and pancreatic insulin content in βAgo2KO animals without any change in islet architecture, α cell number, or total pancreatic mass (Figures 2K–2N, S2I, and S2J). Moreover, no change was detected in TUNEL-positive β cells, indicating cell death was not a primary mechanism for the effect on β cell mass (Figure 2O). Lastly, we measured an increase in both β cell size and the total number of granules (Nv, measured as LDCV volume density), whereas the number of docked granules remained unchanged (Ns, LDCV surface density) in βAgo2KO mice compared to their littermates using electron micrographs (Figure S2K).

### Loss of *miR-184* Expression Promotes β-Cell Proliferation

**Figure 3:**
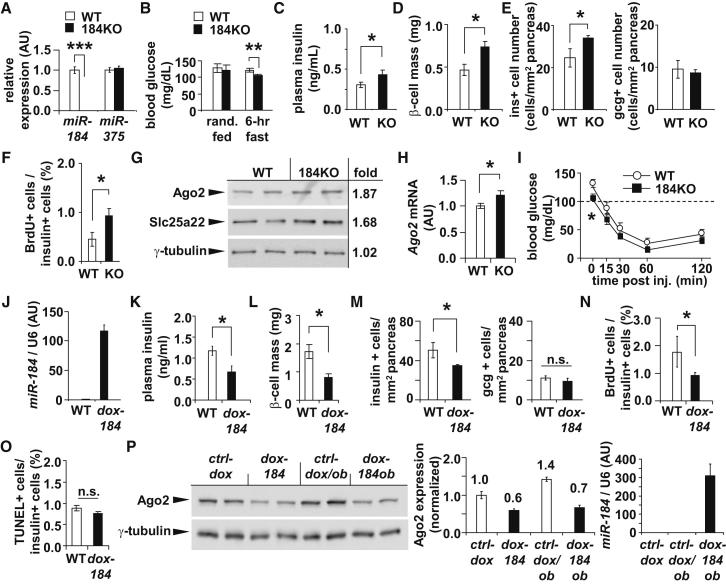
*miR-184* Regulates *Ago2* and Pancreatic β-Cell Proliferation In Vivo (A) Quantitative real-time PCR analysis of *miR-184* and *miR-375* in islets of 10-week-old 184KO mice and wild-type (WT) littermates (n = 4). (B) Blood glucose of 10-week-old 184KO mice and littermates (n = 4). (C) Fasted plasma insulin levels of 10-week-old 184KO mice and littermates (n = 4). (D) β cell mass analysis of 10-week-old 184KO and WT mice (n = 4). (E) Morphometric analysis of insulin+ and glucagon+ cells in 184KO and WT at 10 weeks of age (n = 3). (F) Ratio of BrdU (red) and insulin+ cells (green) in 12-week-old 184KO mice and WT (n = 3). (G) Western blot analysis of Ago2, Slc25a22, and γ-tubulin from islets of 184KO mice and WT. (H) Quantitative real-time PCR analysis of *Ago2* in islets of 10-week-old 184KO mice and littermates (n = 4–5). (I) Blood glucose levels during an ITT on 10-week-old 184KO mice and WT (n = 4–5). (J) Quantitative real-time PCR analysis of *miR-184* in islets of 12-week-old *dox-184* mice and WT mice after 15 days on doxycycline (n = 4). (K) Plasma insulin levels of 10-week-old *dox-184* mice and WT after 15 days on doxycycline (n = 4). (L) β cell mass analysis of 10-week-old *dox-184* mice and WT after 15 days on doxycycline (n = 3). (M) Morphometric analysis of insulin+ and glucagon+ cells in 10-week-old *dox-184* mice and WT (n = 4). (N) Ratio of BrdU and insulin+ cells in *dox-184* mice and WT (n = 4). (O) Ratio of TUNEL+ and insulin+ cells in *dox-184* mice and WT (n = 3). (P) Western blot analysis of Ago2 and γ-tubulin after ex vivo treatment of doxycycline on the islets of *dox-184* and *dox-184ob* mice compared to islets from respective control lean or *ob/ob* littermates. Densitometry is normalized to γ-tubulin expression of *ctrl-dox* islets. Quantitative real-time PCR analysis of *miR-184* in *dox-184ob* mice and *ob/ob* littermates. Results presented as mean ± SEM. ^∗^p < 0.05; ^∗∗^p < 0.01. See also Figure S3.

To determine the functional role of *miR-184* in the β cell, we characterized the constitutive *miR-184*-knockout mouse (184KO) for changes in β cell proliferation, insulin release, and glucose homeostasis. The loss of *miR-184* expression resulted in decreased fasted glucose levels and increased fasted plasma insulin levels (Figures 3A–3C). Consistent with loss of *miR-184* expression during insulin resistance, morphometric analysis of pancreatic sections from 184KO mice showed an increase in β cell mass and number and BrdU incorporation rate (Figures 3D–3F). Moreover, in line with islet expression analysis in insulin-resistant models, Ago2 expression was increased in *miR-184*-deficient islets (Figures 3G and 3H). Knockout mice exhibited transiently elevated insulin release and improved tolerance after glucose challenge, indicating additional targets, such as *Slc25a22*, an established regulator of insulin release, may mediate the effect of *miR-184* on secretion (Figures S1H, S3A, and S3B) (Casimir et al., 2009). Lastly, insulin sensitivity was unchanged in 184KO mice, indicating that loss of this miRNA in the β cell does not contribute to insulin resistance (Figure 3I).

### Restored Expression of *miR-184* in *ob/ob* Mice Inhibits Ago2 and β-Cell Proliferation

**Figure 4:**
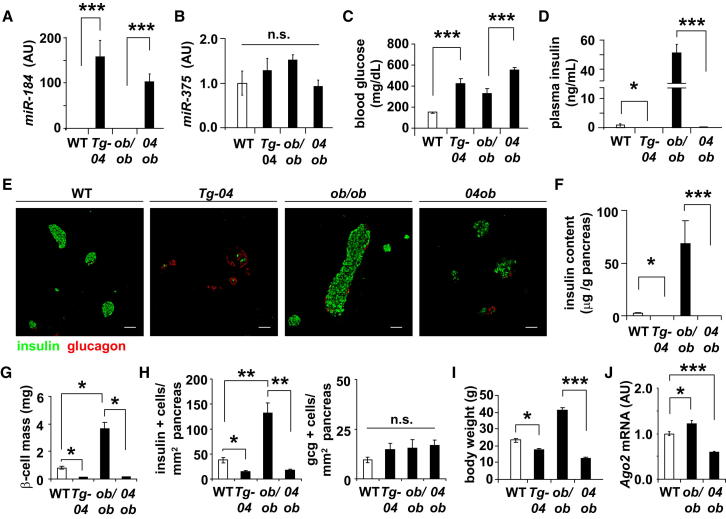
Restoration of *miR-184* during Insulin Resistance Inhibits Compensatory β-Cell Proliferation (A) Quantitative real-time PCR analysis of *miR-184* in islets of 8-week-old wild-type (WT), *Tg-04*, *ob/ob*, and *04ob* mice (n = 3). (B) Quantitative real-time PCR analysis of *miR-375* in islets of 8-week-old WT, *Tg-04*, *ob/ob*, and *04ob* mice (n = 4). (C) Random blood glucose of 8-week-old WT, *Tg-04*, *ob/ob*, and *04ob* mice (n = 4–12). (D) Plasma insulin concentrations of 8-week-old WT, *Tg-04*, *ob/ob*, and *04ob* mice (n = 3–6). (E) Immunostaining analysis of pancreatic sections in 8-week-old WT, *Tg-04*, *ob/ob*, and *04ob* mice for insulin (green) and glucagon (red). Scale bars = 50 μm. (F) Pancreatic insulin content in 8-week-old WT, *Tg-04*, *ob/ob*, and *04ob* mice (n = 4–6). (G) β cell mass analysis of 10-week-old WT, *Tg-04*, *ob/ob*, and *04ob* mice (n = 3). (H) Quantification of insulin+ and glucagon+ cells per area of pancreas in WT, *Tg-04*, *ob/ob*, and *04ob* mice (n = 4). (I) Body weight analysis of WT, *Tg-04*, *ob/ob*, and *04ob* mice (n = 4–12). (J) Quantitative real-time PCR analysis of *Ago2* in islets of 10-week-old WT, *ob/ob*, and *04ob* mice (n = 4–5). Results presented as mean ± SEM. ^∗^p < 0.05; ^∗∗^p < 0.01; ^∗∗∗^p < 0.001. See also Figure S4.

Two gain-of-function models for *miR-184* were then generated in order to restore its expression in the β cell and validate the regulation of *Ago2* by this miRNA during insulin resistance. Similar to *dox-Ago2* mice, Ins2-rtTA mice were crossed to newly generated mice bearing the mouse *miR-184* precursor under regulatory control of a tetracycline-responsive promoter element (*dox-184*). Administration of doxycycline for 15 days via drinking water (1 mg/ml) resulted in ∼100-fold increase in expression (Figure 3J). No change was observed in body weight; however, *dox-184* animals exhibited decreased plasma insulin levels, β cell mass and number, and BrdU incorporation (Figures 3K–3N and S3C). The number of apoptotic cells was unchanged, indicating the hyperglycemia (>250 mg/dl) and glucose intolerance in *dox-184* mice were due to a decrease in β cell mass and rate of proliferation (Figures 3O and S3D–S3F). Isolated islets from *dox-184* mice treated with doxycycline for 48 hr ex vivo exhibited decreased *Ago2* expression (Figure 3P). Moreover, we crossed *dox-184* mice onto the *ob/ob* background (*dox-184ob*) and observed a 50% reduction in Ago2 expression after doxycycline treatment and restoring expression of *miR-184* (Figure 3P). Furthermore, whereas islet levels of *miR-375* and Ago1 were unchanged, random blood glucose levels increased and plasma insulin levels were significantly reduced in *dox-184ob* mice (Figures S3G–S3J). An additional transgenic model of constitutive overexpression was developed to further confirm the role of *miR-184* in β cell growth and function. We focused on three independent mouse lines (*Tg-96*, *Tg-04*, and *Tg-32*), which showed by Southern analysis varying numbers of the transgene containing the *miR-184* precursor sequence under control of the rat *insulin* promoter (Figure S3K). All lines exhibited a mild decrease in body weight, but the highest overexpression of *miR-184* (∼100-fold in *Tg-04* and *32*) resulted in severe hyperglycemia and reduced systemic insulin levels, whereas a 5-fold increase (*Tg-96*) induced glucose intolerance and impaired insulin release (Figures S3L–S3Q). Similar to *dox-184* animals, *Tg-04* and *Tg-32* animals exhibited hyperglycemia and reduced circulating insulin due to loss of β cell mass and pancreatic insulin content (Figures 4A–4G and S4A–S4G). Crossing *Tg-04* animals onto the *ob/ob* background (*04ob*) resulted in sustained expression of *miR-184* and did not compromise the most abundant miRNAs, such as *miR-375* (Figures 4A and 4B). As in *dox-184ob* mice, *04ob* mice exhibited severe hyperglycemia as a result of diminished insulin levels and β cell mass and ultimately contributed to weight loss (Figures 4C–4I). Lastly, restoring expression of *miR-184* in β cells of *04ob* mice suppressed *Ago2* expression compared to *ob/ob* littermates (Figure 4J).

### Argonaute2 Mediates *miR-375* Function

**Figure 5:**
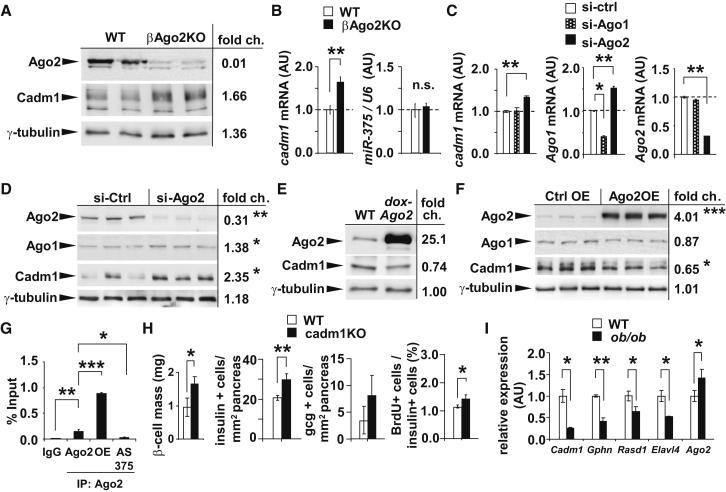
Argonaute2 Mediates *miR-375* Function in the Pancreatic β-Cell (A) Western blot analysis of Ago2 and Cadm1 from islets of 10-week-old βAgo2KO mice and WT littermates. (B) Quantitative real-time PCR analysis of *cadm1* and *miR-375* in islets of 10-week-old βAgo2KO mice and WT littermates (n = 4). (C) Quantitative real-time PCR analysis of *Cadm1*, *Ago1*, and *Ago2* after siRNA-mediated knockdown of *Ago1* and *Ago2* in MIN6 cells (n = 4). (D) Western blot analysis of Ago2, Ago1, and Cadm1 after siRNA-mediated knockdown of *Ago2* compared to scrambled control. (E) Western blot analysis of Ago2 and Cadm1 in islets from 10-week-old *dox-Ago2* mice and WT. (F) Western blot analysis of Ago2, Ago1, and Cadm1 after overexpression of *Ago2* compared to transfection control. (G) Quantitative real-time PCR analysis of *Cadm1* after immunopreciptation of Ago2 from MIN6 cells untransfected (Ago2), after overexpression of Ago2 (OE), and after inhibition of *miR-375* with antisense oligonucleotides (AS375) (n = 4). (H) β cell mass, morphometric analysis of insulin+ and glucagon+ cells, and ratio of BrdU to insulin+ cells in 18-week-old *cadm1*-knockout mice and littermates (n = 4). (I) Quantitative real-time PCR analysis of *Cadm1*, *Gephyrin*, *Rasd1*, *Elavl4*, and *Ago2* in islets of *ob/ob* and WT mice at 16 weeks of age (n = 5–6). Results presented as mean ± SEM. ^∗^p < 0.05; ^∗∗^p < 0.01. See also Figure S5.

We next measured the expression of *miR-375*-targeted genes in islets from βAgo2KO mice and confirmed increased protein expression in vivo (Figure S5A) (Tattikota et al., 2013). Conversely, expression analysis of these *miR-375* targets in islets from *dox-Ago2* mice showed decreased levels by quantitative real-time PCR (Figure S5B). While immunoprecipitation of Ago2 from untransfected MIN6 cells efficiently recovered *miR-375*, we observed an enrichment of *Gephyrin*, *Elavl4/HuD*, and *Rasd1* in the Ago2-containing complexes compared to control pull-downs (Figures S5C and S5D). Moreover, overexpression (OE) of *Ago2* recovered higher levels of these specific mRNAs, whereas transfection of inhibitory antisense oligonucleotides against *miR-375* (AS 375) depleted their presence in the protein complex (Figure S5D). We also observed that expression of the growth suppressor *Cadm1* was increased in islets of βAgo2KO mice, further indicating that the regulation of several targets of *miR-375* was dependent on *Ago2* (Figures 5A and 5B) (Poy et al., 2009). The higher levels of *miR-375* target genes were accompanied by no change in *miR-375* expression, indicating that the loss of Ago2 can nullify the impact of abundant miRNAs on their respective targets (Figure 5B). We next measured *Cadm1* expression after siRNA-mediated knockdown of *Ago2* in MIN6 cells and observed a similar increase, but not after knockdown of *Ago1* (Figures 5C and 5D). In addition, increasing Ago2 levels in both islets of *dox-Ago2* mice and in MIN6 cells (Ago2OE) decreased *Cadm1* expression (Figures 5E and 5F). As observed with the other *miR-375* targets, we detected enrichment of *Cadm1* in Ago2-containing complexes, indicating its regulation was also dependent upon this member of the Ago family (Figure 5G). In line with its established role as a growth suppressor, morphometric analysis in *cadm1*-deficient mice (cadm1KO) showed increased β cell mass and number and BrdU incorporation without any change in total pancreatic mass (van der Weyden et al., 2006, van der Weyden et al., 2012) (Figures 5H and S5E). Lastly, consistent with observations in *dox-Ago2* animals, quantitative real-time PCR analysis in the islets of *ob/ob* mice and littermate controls showed the *miR-375* targets *Cadm1*, *Gephyrin*, *Rasd1*, and *Elavl4/HuD* to be decreased, whereas *Ago2* expression was significantly upregulated (Figure 5I).

### Loss of Argonaute2 Blocks Proliferation during Insulin Resistance

**Figure 6:**
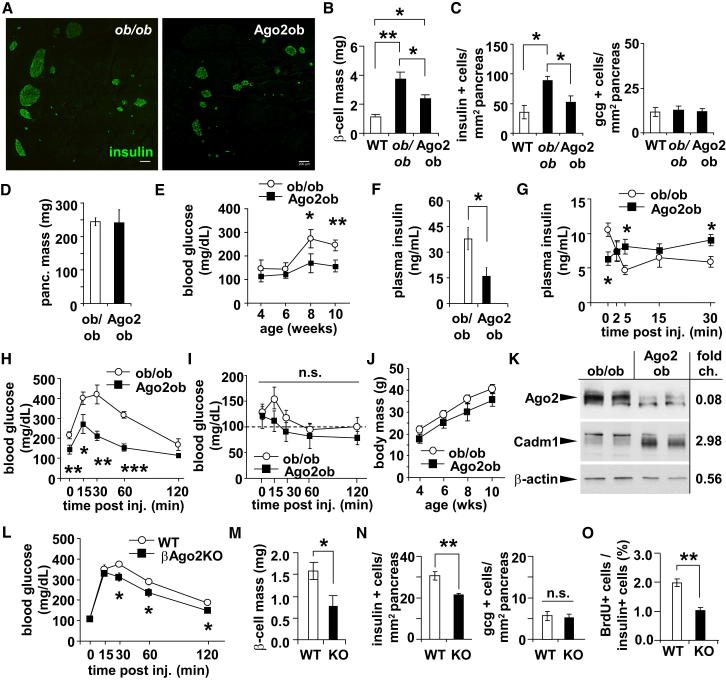
Loss of *Argonaute2* during Insulin Resistance Inhibits Compensatory Proliferation (A) Immunostaining of pancreatic sections from Ago2ob mice and *ob/ob* littermates with antibodies to insulin (green) and glucagon (red). Scale bars = 200 μm. (B) β cell mass analysis of 10-week-old wild-type (WT), *ob/ob*, and Ago2ob mice (n = 3). (C) Morphometric analysis of insulin+ and glucagon+ cells in 10-week-old WT, *ob/ob*, and Ago2ob (n = 4–5). (D) Pancreatic weight in 10-week-old Ago2ob mice and *ob/ob* littermates (n = 4–5). (E and F) Random blood glucose and plasma insulin levels of 10-week-old Ago2ob and littermate *ob/ob* mice (n = 4–5). (G) Plasma insulin levels after glucose bolus on 10-week-old Ago2ob mice and *ob/ob* littermates (n = 4–5). (H) Blood glucose levels during a GTT on 10-week-old Ago2ob mice and *ob/ob* littermates (n = 4–5). (I) Blood glucose levels during an ITT on 10-week-old Ago2ob mice and *ob/ob* littermates (n = 4–5). (J) Body weight of Ago2ob mice and *ob/ob* mice from 4–10 weeks of age (n = 4–5). (K) Western blot analysis of Ago2 and Cadm1 in islets from 10-week-old Ago2ob mice and *ob/ob* littermates. (L) Blood glucose levels during a GTT on 16-week-old βAgo2KO mice and littermates after 12 weeks on a HFD (n = 6). (M) β cell mass analysis in 16-week-old βAgo2KO mice and littermates after 12 weeks on a HFD (n = 6). (N and O) Morphometric analysis of insulin+, glucagon+, and BrdU+ cells in 16-week-old βAgo2KO mice and littermates after 12 weeks on a HFD (n = 6). Results presented as mean ± SEM. ^∗^p < 0.05; ^∗∗^p < 0.01; ^∗∗∗^p < 0.001. See also Figure S6.

To address whether Ago2 mediates the compensatory expansion of β cells during insulin resistance, we next crossed mice carrying the *Ago2*-floxed allele onto the *ob/ob* background to generate mice deficient in both *Ago2* and *leptin* (Ago2ob). In line with βAgo2KO mice, β cell mass and the number of insulin-positive cells were significantly reduced in Ago2ob animals compared to *ob/ob* littermates without any alterations in the number of glucagon-positive cells or total pancreatic mass (Figures 6A–6D). Furthermore, random glucose levels and the corresponding insulin levels were decreased, thereby underlining an important functional role for Ago2 in the growth and function of the β cell (Figures 6E and 6F). In spite of the decrease in β cell mass, Ago2ob mice exhibited higher circulating insulin levels after challenge and improved glucose tolerance, confirming again that loss of *Ago2* increases secretion in vivo (Figures 6G and 6H). Insulin sensitivity, body weight, or food intake (5.2 ± 0.4 in *ob/ob* and 5.0 ± 0.3 g/day in Ago2ob mice) were not significantly altered, but the decrease in both circulating glucose and insulin suggests insulin resistance has been partially attenuated (Figures 6I and 6J). In addition, western blotting analysis of isolated islets showed that loss of Ago2 expression resulted in increased levels of the *miR-375* target *Cadm1* (Figure 6K). Similar results in islet morphometry and glucose tolerance were observed in βAgo2KO mice placed on a HFD, indicating that the effect on β cell mass was consistent in multiple forms of insulin resistance and, although extrapancreatic recombination has been shown with this Cre line, including in the hypothalamus, energy expenditure or food intake was not affected (Figures 6L–6O and S6A–S6D).

### Administration of the Ketogenic Diet to *ob/ob* Mice Restores *miR-184* Expression

**Figure 7:**
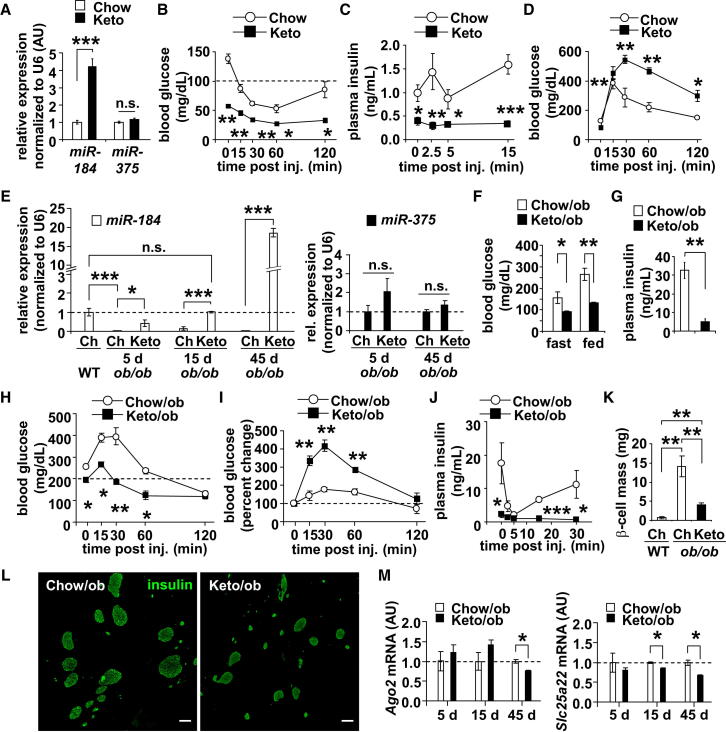
Administration of the Ketogenic Diet during Insulin Resistance Rescues *miR-184* Expression (A) Quantitative real-time PCR analysis of *miR-184* and *miR-375* in islets of 10-week-old C57BL/6 mice on chow (Chow) or ketogenic diet (Keto) for 24 days (n = 4). (B) Blood glucose levels during an ITT on 10-week-old Chow and Keto mice for 24 days (n = 4–5). (C and D) Plasma insulin and blood glucose levels after glucose challenge on 10-week-old Chow and Keto mice for 24 days (n = 4–5). (E) Quantitative real-time PCR analysis of *miR-184* and *miR-375* in islets of 16-week-old *ob/ob* mice on chow or ketogenic diet and WT littermates (n = 4). (F and G) Blood glucose and random plasma insulin levels in 16-week-old *ob/ob* mice on chow (Chow/ob) or ketogenic diet (Keto/ob) for 15 days (n = 4). (H) Blood glucose levels during an ITT on 16-week-old *ob/ob* mice on chow or ketogenic diet for 15 days (n = 4–5). (I and J) Plasma insulin and blood glucose levels after glucose challenge on 16-week-old *ob/ob* mice on chow or ketogenic diet for 15 days (n = 4-5). (K and L) Quantification of β cell mass after immunostaining pancreatic sections for insulin (green) of 16-week-old *ob/ob* mice on chow or ketogenic diet for 45 days (n = 3). Scale bars = 200 μm. (M) Quantitative real-time PCR analysis of *Ago2* and *Slc25a22* in islets of 16-week-old *ob/ob* mice on chow or ketogenic diet (n = 4–5). Results presented as mean ± SEM. ^∗^p < 0.05; ^∗∗^p < 0.01; ^∗∗∗^p < 0.001. See also Figure S7.

Published studies have shown that the administration of the ketogenic diet improves insulin sensitivity and glycemic status (Badman et al., 2009). To further support the functional relevance of *miR-184* and *Ago2* in the β cell, we next tested whether *miR-184* is regulated after administering the ketogenic diet to C57BL/6 mice (Keto mice). After 24 days on the diet, we observed ∼4-fold increase of *miR-184* in islets from Keto mice (Figure 7A). As previously shown, Keto mice exhibited decreased body weight and reduced glucose levels at steady state and during an ITT in line with improved insulin sensitivity (Figures 7B, S7A, and S7B). Consistent with mild ∼4-fold, overexpression of *miR-184*, Keto mice exhibited reduced insulin levels and impaired tolerance compared to control mice after a glucose challenge; however, no change was observed in β cell mass (Figures 7C, 7D, and S7C). Moreover, administration of the ketogenic diet to *ob/ob* mice (Keto/*ob)* for 15 days was sufficient to restore normal expression of *miR-184*; however, after 45 days, we observed a ∼18-fold increase in the expression of this miRNA in islets from Keto/*ob* mice compared to chow-fed, nonobese littermate controls (Ch-WT) (Figure 7E). Keto/*ob* mice also exhibited lower glucose levels during an ITT, reduced random glucose and plasma insulin levels, and no change in body weight (Figures 7F–7H, S7D, and S7E). Similar to Keto mice, Keto/*ob* mice were relatively more glucose intolerant as a result of decreased insulin release compared to Chow/ob littermates (Figures 7I and 7J). These results may reflect a significant reduction in the demand for insulin because Keto/*ob* mice are normoglycemic. Moreover, after 45 days on the ketogenic diet, we observed a decrease in β cell mass and Ki-67+ β cells in Keto/*ob* animals, and quantitative real-time PCR analysis at this time point showed *Ago2* and *Slc25a22* expression to be significantly reduced, whereas measurements produced by western blotting showed Cadm1 to be increased (Figures 7K–7M, S7F, and S7G). Taken together, these data further show that both *miR-184* and *Ago2* expression are inversely regulated in accordance with changes in the requirement for insulin.

## Discussion

Here, we show that the onset of insulin resistance induces the silencing of *miR-184* in order to promote the function of *Ago2* in the pancreatic β cell. Our data suggest that *miR-184* acts as a “brake” on *Ago2* that becomes released to increase proliferation and accommodate the elevated demand for insulin. Moreover, we show that the increased expression of Ago2 will facilitate the function of *miR-375* in suppressing genes, including *Cadm1* in vivo. Our observations functionally associating *miR-184* together with *Ago2* and *miR-375* now identify a network within the miRNA pathway that coordinately regulates the compensatory proliferation of the β cell.

Interestingly, *miR-184* is unique as the most downregulated islet miRNA during insulin resistance, and additionally, administration of the ketogenic diet in *leptin*-deficient *ob/ob* mice rescued its expression. Furthermore, the increase of *miR-184* expression in the islets of *ob/ob* mice after restoring insulin sensitivity further underlines a dynamic role for the miRNA pathway in regulating β cell growth and function according to the demand for insulin. Published work identified the regulation of miRNAs in the retina according to light levels, thereby linking turnover of small RNAs with physiologic stimuli in neuronal cells; together, our results may indicate another common property between these cell types (Krol et al., 2010). Likewise, after treatment with serotonin in *Aplysia* sea snails, *miR-184* was significantly downregulated in the nervous system, the site of orthologous insulin expression (Rajasethupathy et al., 2009, Floyd et al., 1999). Moreover, serotonin has been previously shown to mediate compensatory expansion of the β cell during pregnancy (Kim et al., 2010). In line with these studies, we observed that *miR-184* was transiently decreased at day 18.5 of gestation in C57BL/6 mice and that the expression of this miRNA had been restored by day 5 in the postpartum phase. Neither *tryptophan hydroxlase 1* nor *2* (*Tph1*/*2*) were regulated in the pancreatic islets of *ob/ob* mice, further suggesting that multiple independent pathways promote proliferation in these models (Rieck et al., 2009). However, the robust consistency of the silencing of *miR-184* in both models of pregnancy and insulin resistance suggests that additional genes in the miRNA pathway will be identified as mediators of β cell proliferation (Jacovetti et al., 2012).

Importantly, our results continue to build on the numerous studies now supporting a role for miRNAs in mediating cellular stress responses (Leung and Sharp, 2010, Mendell and Olson, 2012). The observations made in Ago2ob animals would continue to indicate that several abundant miRNAs in the β cell coordinately contribute to this process. In addition, our current investigation continues to reinforce the role of miRNAs as inhibitors of insulin release (Poy et al., 2004, Tattikota et al., 2013, Bolmeson et al., 2011). Although the precise role of miRNAs remains unclear, the accumulating evidence may suggest that “brakes” on gene expression are essential in managing the dynamic nature of both first- and second-phase insulin release (Wang and Thurmond, 2009). Of note, the increased release of insulin after loss of *Ago2* expression in the β cells of *ob/ob* mice improved many aspects of the diabetic phenotype, including circulating glucose and insulin levels, and glucose tolerance; however, this came at the expense of β cell mass and may suggest that the miRNA pathway mediates a delicate balance between both growth and secretion. Many of the genes being targeted by small RNAs in the β cell may regulate this balance via independent mechanisms. Several studies indicate F-actin and the microtubule networks are actively engaged in managing insulin release and many of the targets of *miR-375*, including *Cadm1* and *Gephyrin*, may contribute to the remodeling of the cytoskeleton (Seino et al., 2011, Charrier et al., 2010, Masuda et al., 2010). In addition, published studies have already shown that during pregnancy, Rasd1 acts as a growth suppressor and is inhibited during islet expansion and then is increased in the postpartum phase when islet mass is restored (Lellis-Santos et al., 2012). Also, inhibition of the RNA-binding protein HuD/Elavl4 was shown to promote expression of insulin (Lee et al., 2012). Lastly, as evidenced in the differences in the phenotypes of *dox-184* and βAgo2KO mice, our observations supporting *Slc25a22*, a regulator of glutamate transport and secretion in the β cell, as an additional target of *miR-184* indicates that this miRNA may also regulate both proliferation and exocytosis through the targeting of different genes (Casimir et al., 2009). Future studies will presumably continue to establish that numerous targets of all miRNAs conspire to orchestrate the demands placed on the cell during insulin resistance, including growth and the continual recruitment of insulin-containing granules. It will also remain to be seen whether specific miRNA-mediated gene regulations are unique to their metabolic context, including insulin resistance, fasting, hypoxia, and cold exposure (Dumortier et al., 2013). Published studies have illustrated reduced expression of *miR-375* in the pancreatic islets of *db/db* mice and Goto-Kakizaki rats, suggesting that differential roles may exist for miRNAs and their targets according to alterations in the metabolic environment (Zhu et al., 2013, El Ouaamari et al., 2008).

Therefore, it will be important to understand the factors that dictate the kinetics of miRNA regulation in the β cell. It also remains to be determined whether the same genes that expedite the decay of *miR-184* during insulin resistance are the same factors that promote its expression as sensitivity improves after administration of the ketogenic diet. Moreover, the increase in *miR-184* that results from this diet and the resulting suppression of insulin release indicate that miRNAs play a significant role in the maintenance of steady-state circulating glucose and insulin levels. Furthermore, the identification of increased *miR-184* expression can explain the simultaneous occurrence of both improved insulin sensitivity and glucose intolerance observed in mice on ketogenic diet. Although the results appear paradoxical at first, this may be explained by the drastic reduction in both circulating glucose and the demand for insulin. Another apparent conflict appears in the mouse models exhibiting hypersecretion and decreased β cell mass. However, in spite of the reduction in β cell mass in Ago2ob mice (35% lower compared to *ob/ob* littermates), the decrease is significantly below the threshold that would induce hyperglycemia (Orland et al., 1985). *Ago2*-deficient models still maintain the capacity to transiently secrete higher amounts of insulin because β cells release a relatively small percentage of its stored granule pool in response to glucose (Seino et al., 2011). Conversely, although increased expression of Ago2 causes transient glucose intolerance in *dox-Ago2* mice, these mice have normal circulating glucose and insulin levels and insulin sensitivity, and therefore it is unlikely that the increase in Ago2 levels in the islets of *ob/ob* mice plays a causal role in the hyperglycemia or hyperinsulinemia. As shown in *dox-184ob* animals, restoring *miR-184* in the β cell and reducing Ago2 levels in an insulin-resistant model exacerbated the diabetic phenotype. In contrast, reducing glucose levels and the demand for insulin in *ob/ob* mice by administration of ketogenic diet to ultimately suppress *Ago2* and restore β cell mass underlines an important function this gene plays in the adaptation of the β cell according to metabolic need.

Many aspects of β cell physiology remain largely unexplored in terms of nutrient sensing, cellular metabolism, and intercellular signaling pathways. Our observations on the effects of the ketogenic diet on miRNA function in the β cell unify several poorly understood mechanisms and reinforce the potential in studying the role of small RNAs in physiologic stresses. Continued emphasis on characterizing the functional role of miRNA targets in this cell type should bring clarity in understanding how the network of small RNAs contribute to maintaining essential metabolic processes and how their failure ultimately leads to disease.

## Experimental Procedures

### Generation and Maintenance of Animals

Mice were maintained on a 12 hr light/dark cycle with ad libitum access to regular chow food, a HFD (containing 60% kcal fat, cat. no. E15741-347, ssniff Spezialdiäten GmbH), or a ketogenic diet (cat. No. E15149-30, ssniff Spezialdiäten GmbH) in accordance with requirements established by Landesamt für Gesundheit und Soziales (Lageso). All experimental procedures were approved under protocols G 0357/10, O 0405/09, and T 0436/08. Results were consistent in both genders; however, data from female mice are not shown. Total *Cadm1* knockouts and floxed *Ago2* mice were generated as described (van der Weyden et al., 2006, O’Carroll et al., 2007). The total *miR-184* knockout was generated by D. Aberdam and R. Shalom-Feuerstein (Institut Clinique de la Souris) and genotyped with forward and reverse primers 5′ ACTGAACATTATTTCATGGGCCGGG and 5′ AACTACAACTGTTTGGCTAGCAGGGTG (knockout allele) and the substitute reverse primer 5′ CGCTGAGACCTTGTGATAAACCGTT (wild-type allele). To generate *dox-184* mice, a 292 bp genome fragment encompassing *miR-184* precursor was amplified using the primers 5′ TGCTGAAGAGTGGCCTGCTAGG and 5′ CTCCTCCTCACGTCCTGTGGTA, cloned into a pTRE-2 vector (Clontech), and the resulting construct was used for microinjections. The *Ago2*-tetO mice were generated from a mouse *Ago2* cDNA construct (a kind gift of G. Meister, University of Regensburg) (Nir et al., 2007).

### Gene Expression Analysis in Mouse and Human Islets

Small RNA sequencing and expression analysis from cell lines and mouse islets was performed as described; primers used with FastStart SYBR Green PCR Master Mix (Roche) are available upon request (Tattikota et al., 2013). Relative quantification of *Ago* genes was determined after normalization with standard curves established with mouse *Ago1-4* cDNA constructs (G. Meister). RIP experiments using Ago1 and Ago2 antibodies were performed as described (Keene et al., 2006). Human islets were obtained with research consent and selected based on islet cell purity and isolated at the University of Virginia, Oxford DRWF Human Islet Isolation Facility and Prodo Laboratories (USA) (Morán et al., 2012). Information for nondiabetic and diabetic donors is provided in Table S2.

### Analytic Procedures

Insulin measurements from plasma and pancreatic extracts were measured by radioimmunoassay (RIA) (Millipore), and blood glucose and luciferase assays were measured as described previously (Poy et al., 2009). Islet morphometric analysis after intraperitoneal injections of BrdU on four consecutive days (50 μg/g BW, Sigma B5002) was performed on 8 μm sections of paraffin-embedded pancreas approximately 150–200 μm apart. Sections were dewaxed, washed, and stained for insulin (Dako A0564), glucagon (Millipore AB932), BrdU (Abcam ab6326), Ki-67 (Dako), or TUNEL (Roche cat. no.12156792910). Cell numbers from all islets in 3–7 sections were counted with ImageJ software from 20X images obtained using a Zeiss LSM700 (Schneider et al., 2012). β cell mass was measured as the ratio of insulin-positive cell area to the total tissue area, multiplied by the weight of the pancreas using Imaris software (Bitplane). In vivo insulin release and glucose (GTT) or insulin (ITT) tolerance tests were performed following a 6 hr fast and injected intraperitoneally with either glucose (2 g/kg BW) or insulin (0.75 U/kg BW).

### Cell Culture, Immunoprecipitation, and Antibodies

MIN6 cells were cultured in Dulbecco’s modified Eagle’s medium (Invitrogen) containing 4.5 g/l glucose supplemented with 15% v/v heat-inactivated FCS, 50 μM β-mercaptoethanol, and 50 mg/ml penicillin, and 100 mg/ml streptomycin and insulin release was performed as described (Poy et al., 2004). Antibodies were as described: Gephyrin (BD 610584), Rasd1 (Millipore AB15794), Ago2 (for immunoprecipitation, Wako 018-22021), Ago2 (for western blotting, Cell Signaling C34C6), Ago1 (MBL RN028PW), Slc25a22 (Sigma AV44041), HuD/Elavl4 (Santa Cruz sc-48421), γ-tubulin (Sigma T6557), Cadm1 (Sigma S4945), and β-actin (Sigma). Guinea pig anti-insulin and rabbit anti-glucagon antibodies (Millipore) were used on paraffin-embedded pancreata fixed in 4% paraformaldehyde for 3 hr.

### Statistical Analysis

Pearson’s R coefficient and independent two-group Mann-Whitney tests were implemented using the R statistical package (http://www.r-project.org). Comparisons between data sets with two groups were evaluated using an unpaired Student’s t test. ANOVA analysis was performed for comparisons of three or more groups. A p value of less than or equal to 0.05 was considered statistically significant. Results presented as mean ± SEM. ^∗^p < 0.05; ^∗∗^p < 0.01; ^∗∗∗^p < 0.001.

## Accession Numbers

The NCBI Gene Expression Omnibus (GEO) accession number for referenced array data is GSE46623.

## Supplemental Information

Document S1. Figures S1–S7, Supplemental Experimental Procedures, and Table S2

Table S1. Small RNA Sequencing Profile of Pancreatic Islets from *ob/ob* Mice and C57BL/6J Littermates, Related to Figure 1
