# Supplementary material for: Argonaute2 Mediates Compensatory Expansion of the Pancreatic β Cell
Source: Cell Metab. 2014 Jan 7;19(1):122–34. doi: 10.1016/j.cmet.2013.11.015 (PMC3945818; doi:10.1016/j.cmet.2013.11.015)
Supplement: Document S1. Figures S1–S7, Supplemental Experimental Procedures, and Table S2 [file mmc1.pdf]

Cell Metabolism, Volume *19*

Supplemental Information

Argonaute2 Mediates Compensatory Expansion of the Pancreatic β Cell

Sudhir G. Tattikota, Thomas Rathjen, Sarah J. McAnulty, Hans-Hermann Wessels, Ildem Akerman, Martijn van de Bunt, Jean Hausser, Jonathan L.S. Esguerra, Anne Musahl, Amit K. Pandey, Xintian You, Wei Chen, Pedro L. Herrera, Paul R. Johnson, Donal O'Carroll, Lena Eliasson, Mihaela Zavolan, Anna L. Gloyn, Jorge Ferrer, Ruby Shalom-Feuerstein, Daniel Aberdam, and Matthew N. Poy

Figure S1, related to Figure 1.

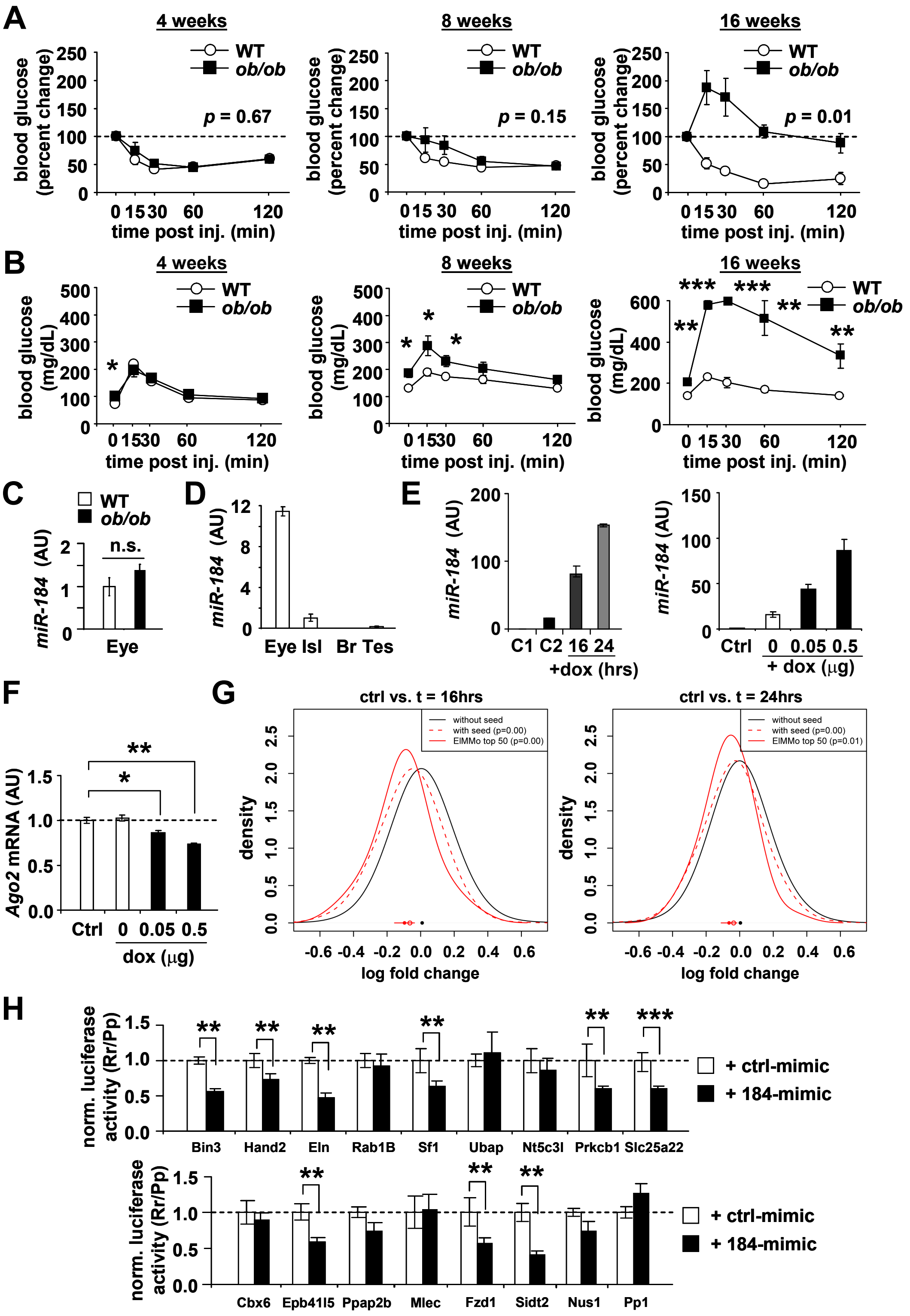

Figure S2, related to Figure 2

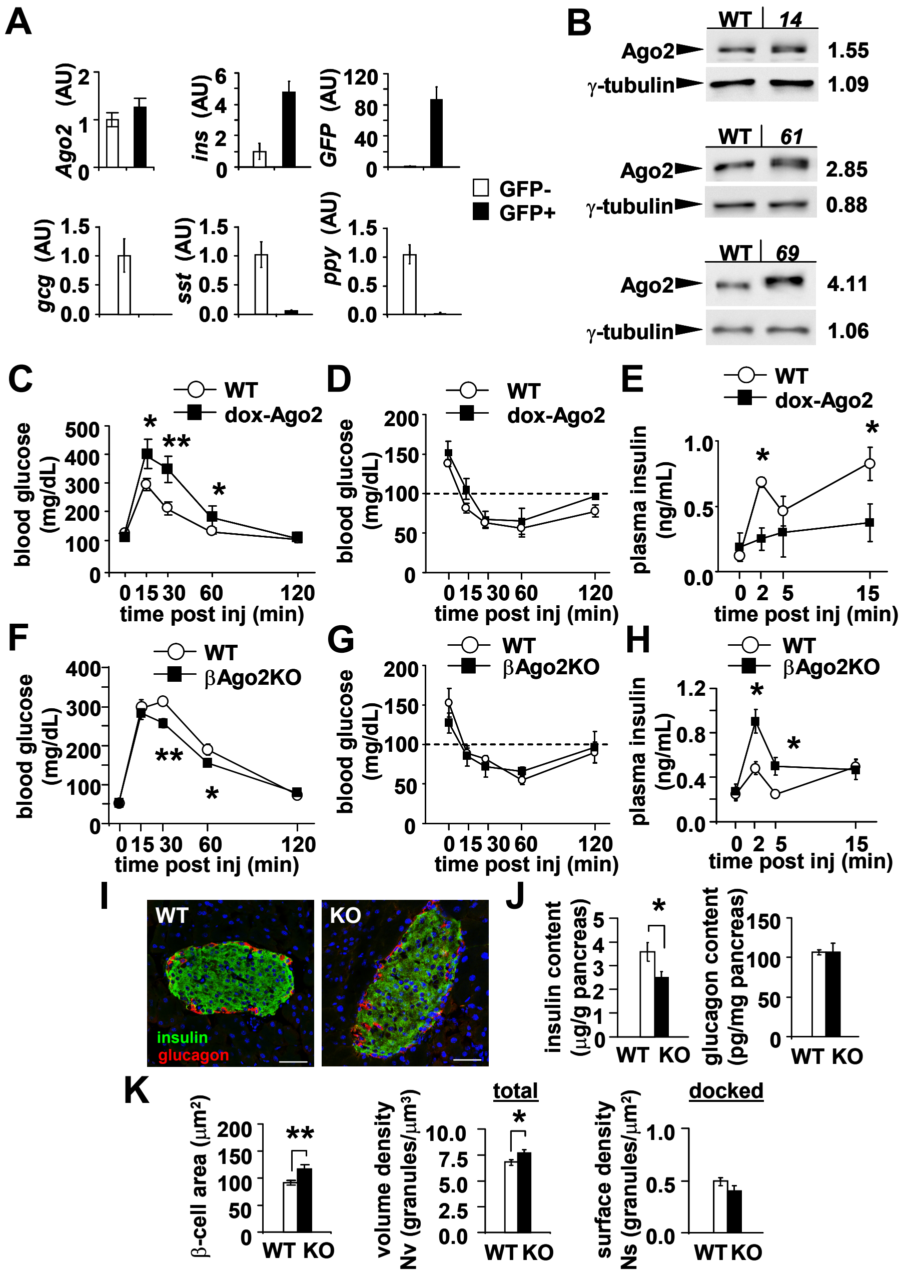

Figure S3, related to Figure 3.

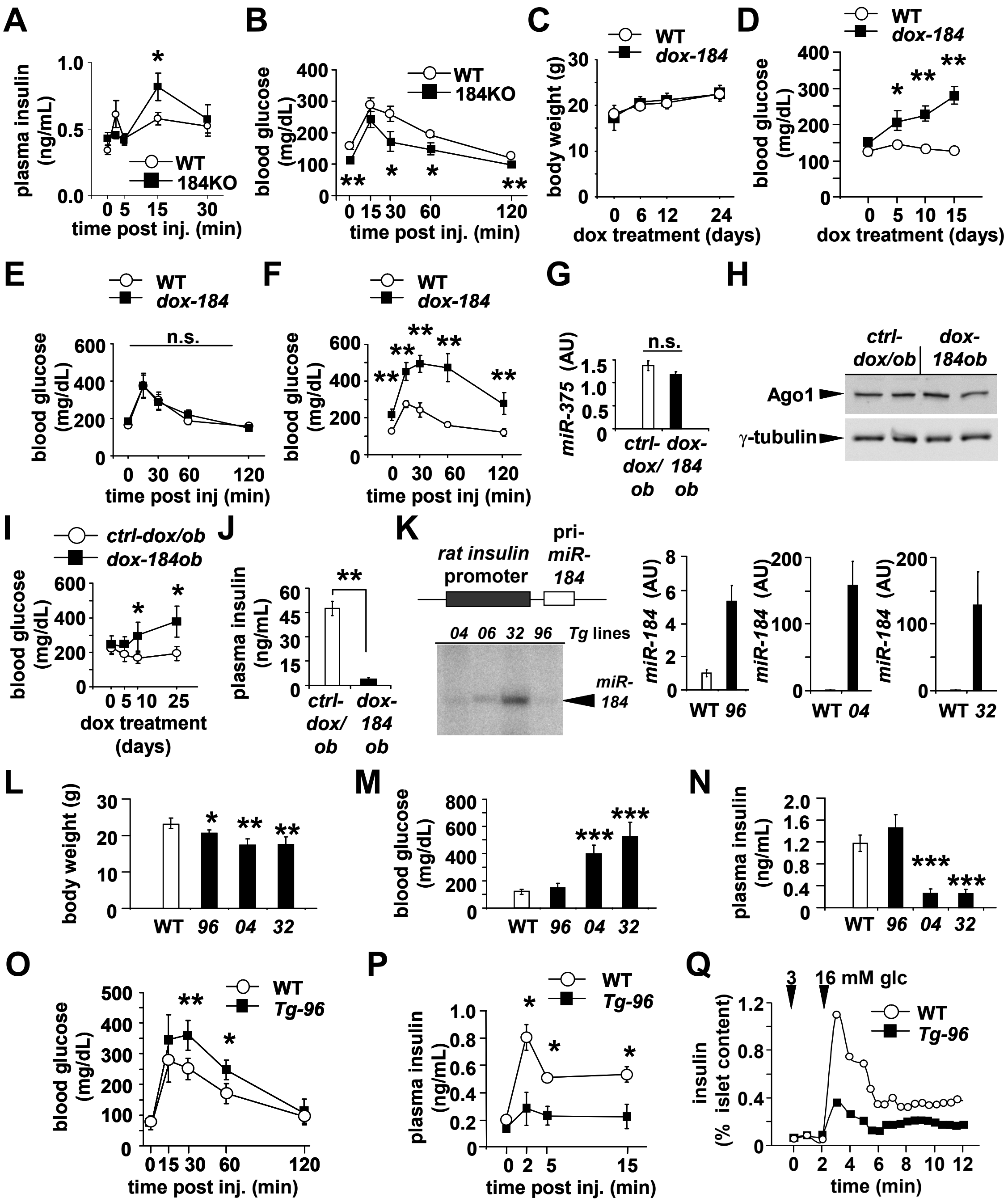

Figure S4, related to Figure 4.

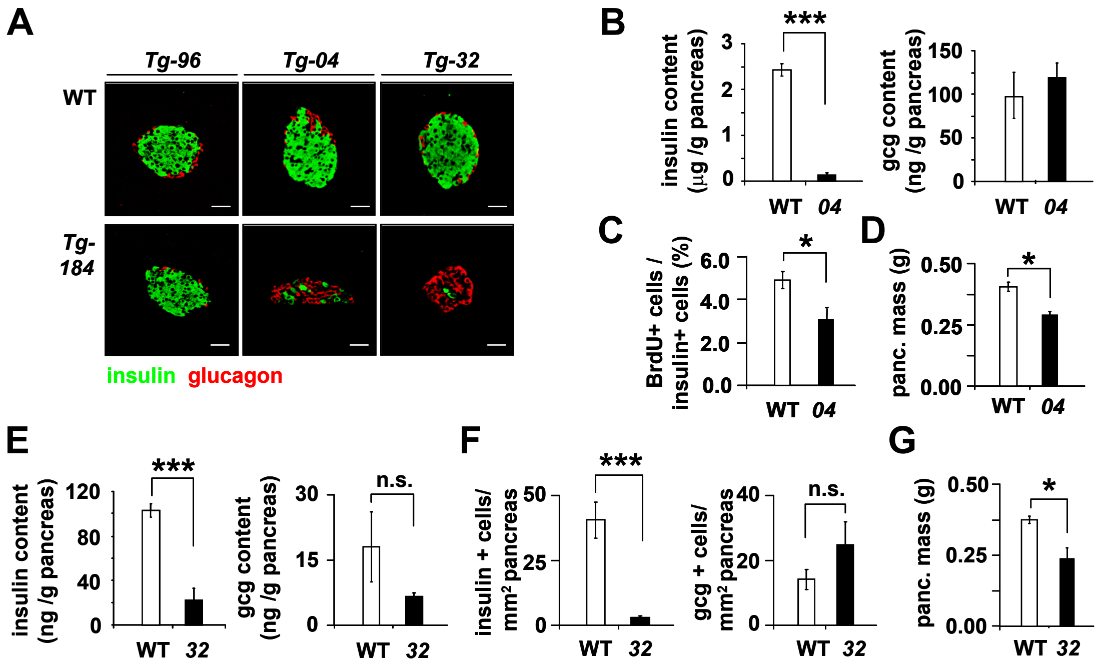

Figure S5, related to Figure 5.

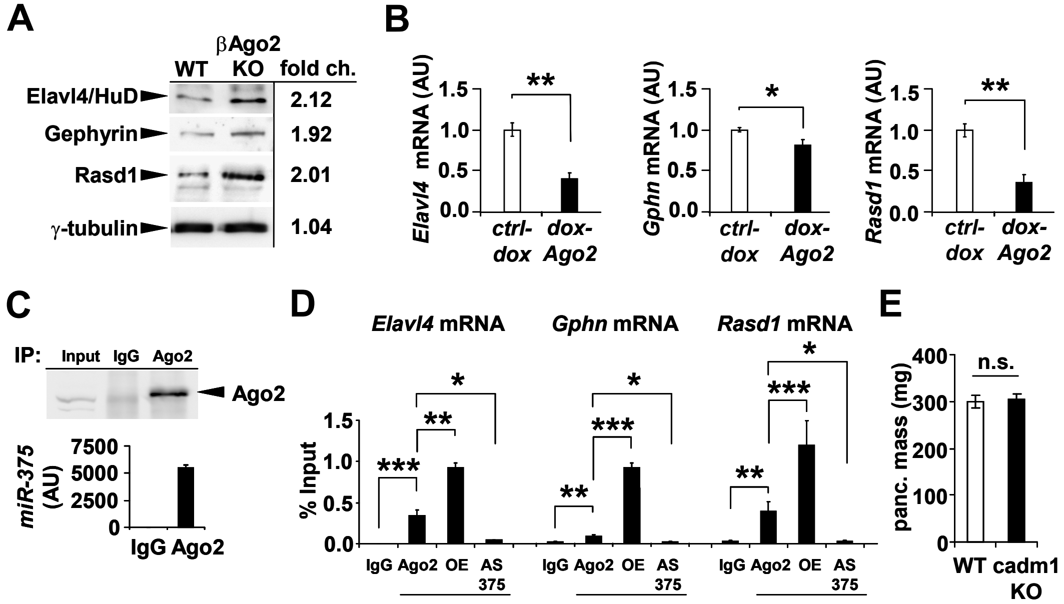

Figure S6, related to Figure 6.

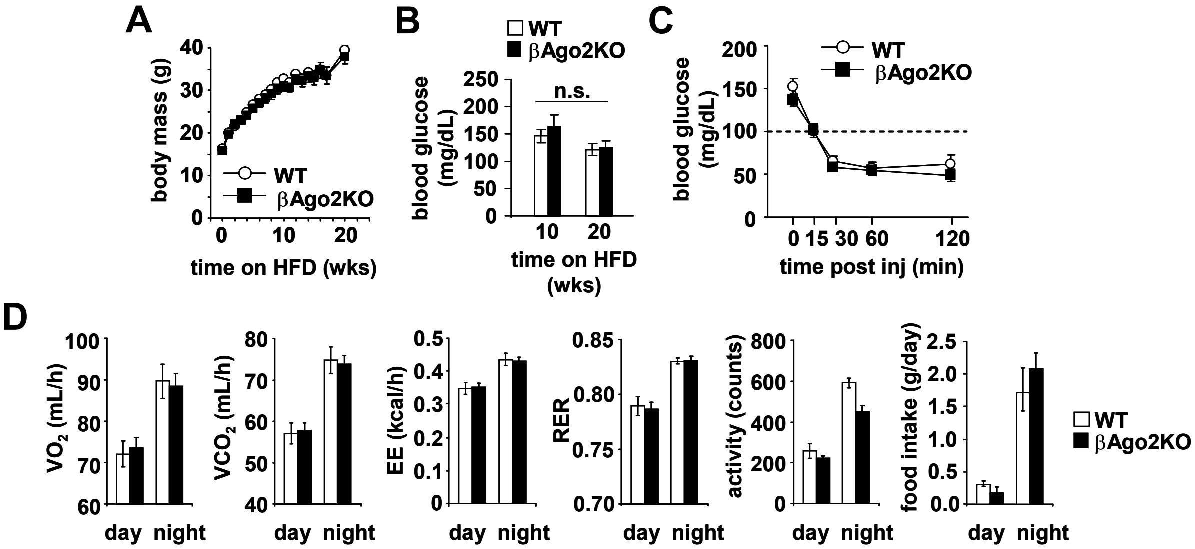

Figure S7, related to Figure 7.

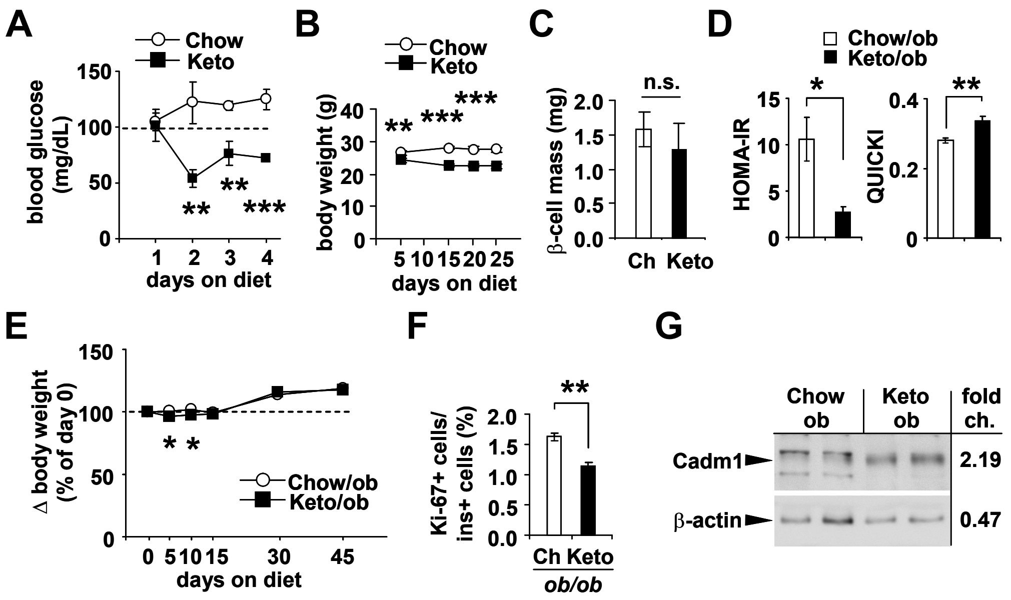

Supplemental Figure Legends

Figure S1. Relative expression of *miR-184* in different tissues and the identification of its putative targets, related to Figure 1. (A) Blood glucose measurements during an insulin tolerance test on *ob/ob* mice and control littermates, *P* value derived from mean of area under the curve (n=3-4). (B) Blood glucose measurements during a glucose tolerance test (1g glucose/kg BW) on *ob/ob* mice and control littermates (n=3-4). (C) qRT-PCR analysis of *miR-184* in the eye of *ob/ob* mice and littermate controls at 16 weeks of age (n=3). (D) qRT-PCR analysis of *miR-184* in eye, islets, brain and testes of 12-week old C57BL/6 mice (n=3). (E) qRT-PCR analysis of *miR-184* in MIN6 cells transfected with plasmids expressing the rtTA transactivator and *miR-184* under control of the operator sequence of the *E.coli* tetracycline-resistance operon (184-tetO) after treatment of doxycycline; (C1) untransfected control (C2) untreated control (n=3). (F) qRT-PCR analysis of *Ago2* after inducing expression of *miR-184* with doxycycline (n=3). (G) Probability density function of log2 fold changes in transcript abundance after inducing *miR-184* showing that transcripts with a *miR-184* motif (dashed red line) or an evolutionarily-conserved *miR-184* motif (solid red line) are down-regulated after induced over-expression from *184-tetO* construct relative to transcripts that do not contain this motif. (H) Luciferase assays in HEK293 cells testing computationally-predicted target genes of *miR-184* after transfection of microRNA mimic (184-mimic) or scrambled control (ctrl-mimic). Results are presented as mean ± SEM. *p<0.05, and **p<0.01.

Figure S2. *Argonaute2* regulates insulin release *in vivo,* related to Figure 2. (A) qRT-PCR analysis of *Argonaute2 (Ago2)*, *insulin* (*ins*), *green fluorescent protein* (*GFP*), *glucagon* (*gcg*), *somatostatin* (*sst*), and *pancreatic polypeptide* (*ppy*) of pancreatic β-cells isolated after FACS-sorting from 12-week old mouse *insulin* promoter-*GFP* mice (n=3). (B) Western blot analysis of Ago2 from isolated islets of 10-week old *dox-Ago2* mice (lines 14, 61, and 69) and WT. (C) Blood glucose during a glucose tolerance test (GTT) on 10-week old *dox-Ago2* mice (line 30) and WT (n=4-5). (D) Blood glucose during an insulin tolerance test (ITT) on 10-week old *dox-Ago2* mice (line 30) and controls (n=4-5). (E) Plasma insulin levels after glucose infusion on 10-week old *dox-Ago2* mice and littermates (n=5). (F) Blood glucose during a GTT on 10-week old βAgo2KO mice and WT (n=4-5). (G) Blood glucose during an ITT on 10-week old βAgo2KO mice and littermates (n=3). (H) Plasma insulin levels after glucose infusion on 10-week old βAgo2KO mice and WT (n=5). (I) Immunostaining of pancreatic sections from 10-week old βAgo2KO mice and littermates with antibodies to insulin (green) and glucagon (red). Scale bars, 50 μm. (J) Total pancreatic insulin and glucagon content in 10-week old βAgo2KO and littermates (n=5-8). (K) Quantification of cell area (μm2) on random β-cells in βAgo2KO and WT from 10 weeks of age (n=5-8). Quantification of docked large dense core vesicles (LDCVs) measured as surface density Ns (granules/μm^2) and the total amount of LDCVs measured as volume density Nv (granules/μm^3) in βAgo2KO and WT from 10 weeks of age (n=3). Results are presented as mean ± SEM. *p<0.05, and **p<0.01.

Figure S3. *miR-184* regulates glucose homeostasis *in vivo*, related Figure 3. (A) Plasma insulin levels after glucose challenge (2g glucose/kg BW) in 10-week old 184KO mice compared to control mice (n=3-4). (B) Blood glucose levels during intraperitoneal glucose tolerance test (GTT) (2g glucose/kg BW) in 10-week old 184KO mice compared to control mice (n=3-4). (C) Body weight analysis of 10-week old *dox-184* transgenic

mice compared to control mice after 24 days on doxycycline (1 mg/ml) (n=4). (D) Random blood glucose measurements of 10-week old *dox-184* transgenic mice compared to control mice after receiving doxycycline (1 mg/ml) (n=6-8). (E,F) Blood glucose levels during intraperitoneal glucose tolerance test (GTT) (1g glucose/kg BW) in 10-week old *dox-184* mice compared to control mice before and after 24 days on doxycycline (1 mg/ml) (n=4). (G) qRT-PCR analysis of *miR-375* in *dox-184ob* mice and control *ob/ob* littermates. (H) Western blotting analysis of Ago1, and γ-tubulin after ex vivo treatment of doxycycline on islets from *dox-184/ob* mice compared to islets from respective control *ctrl-dox/ob* littermates. (I) Random blood glucose measurements of 10-week old *dox-184ob* mice compared to *ob/ob* littermate controls (n=4). (J) Plasma insulin levels of 10-week old *dox-184ob* mice and controls after 15 days on doxycycline (n=4). (K) Schematic representation of the *miR-184* transgene construct. The mouse *miR-184* precursor sequence was positioned downstream of the rat *Ins2* promoter and Southern analysis estimated the number of transgene insertions. RT-PCR analysis of *miR-184* in isolated pancreatic islets of 12-week old *Tg-96*, *Tg-04*, and *Tg-32* transgenic mice compared to control mice (n=4). (L) Body weight analysis of 10-week old *Tg-96*, *Tg-04*, and *Tg-32* transgenic mice compared to control mice (n=6-8). (M) Random blood glucose measurements of 10-week old *Tg-96*, *Tg-04*, and *Tg-32* transgenic mice compared to control mice (n=6-8). (N) Plasma insulin concentrations of 10-week old *Tg-96*, *Tg-04*, and *Tg-32* transgenic mice compared to control mice (n=6-8). (O) Blood glucose levels during intraperitoneal glucose tolerance test (GTT) (2g glucose/kg BW) in 10-week old *Tg-96* transgenic mice compared to control mice (n=5-6). (P) Plasma insulin levels after glucose challenge (2g glucose/kg BW) in 10-week old *Tg-96* transgenic mice compared to control mice (n=5-6). (Q) Insulin measurements over time during perifusion analysis in isolated islets from 10-week old *Tg-96* transgenic mice compared to islets from control mice. Results are presented as mean ± SEM. *p<0.05, and **p<0.01.

Figure S4. Transgenic over-expression of *miR-184* decreases pancreatic β-cell mass, related to Figure 4. (A) Immunostaining analysis of pancreatic sections in 10-week old *Tg-96*, *Tg-04*, and *Tg-32* transgenic mice compared to control mice with antibodies to insulin (green) and glucagon (red). Scale bars, 50 μm. (B) Total pancreatic insulin and glucagon content in 10-week old *Tg-04* and *Tg-32* transgenic mice compared to control mice. (C) Quantification of BrdU-positive cells per insulin-positive cells in *Tg-04* mice compared to littermate control animals (n=4). (D) Quantification of pancreatic mass in *Tg-04* mice compared to littermate control animals (n=6). (E) Total pancreatic insulin and glucagon content in 10-week old *Tg-32* transgenic mice compared to control mice. (F) Quantification of insulin and glucagon-positive cells per area of pancreas in *Tg-32* mice compared to littermate control animals (n=4). (G) Quantification of pancreatic mass in *Tg-32* mice compared to littermate control animals (n=6). Results are presented as mean ± SEM. *p<0.05, **p<0.01 and ***p<0.001.

Figure S5. Argonaute2 mediates suppression of targets by *miR-375*, related to Figure 5. (A) Western blotting analysis of *miR-375* targets from isolated pancreatic islets of 10-week old βAgo2KO mice and littermate controls. (B) qRT-PCR analysis of *Gephyrin, Rasd1* and *Elavl4* in isolated pancreatic islets of 10-week old *dox-Ago2* mice and littermate controls (n=4). (C) Detection of Argonaute2 after immunoprecipitation from MIN6 cell lysates with α-Ago2 antibody and quantification of *miR-375* by qRT-PCR after isolation of total RNA from Ago2-associated complexes. (D) qRT-PCR analysis of *Elavl4, Gephyrin,* and *Rasd1* after immunopreciptation of Ago2 from MIN6 cells either untransfected (Ago2), after over-expression of Ago2 (OE), and after inhibition of *miR-*

375 with antisense oligonucleotides (AS375) (n=4). (E) Quantification of pancreatic mass in cadm1KO mice compared to littermate control animals (n=5). Results are presented as mean ± SEM. *p<0.05, and **p<0.01.

Figure S6. βAgo2KO mice on high fat diet do not exhibit changes in body weight or energy expenditure, related to Figure 6. (A) Body weight quantification of 24-week old βAgo2KO and control littermate mice after 20 weeks on high fat diet (n=6). (B) Random blood glucose measurements of βAgo2KO and control littermate mice after 10 and 20 weeks on high fat diet (n=6). (C) Blood glucose measurements during an insulin tolerance test on βAgo2KO and control littermate mice after 20 weeks on high fat diet. (D) Quantification of VO2, VCO2, energy expenditure (EE), activity and food intake in βAgo2KO and control littermate mice after 20 weeks on high fat diet. Results are presented as mean ± SEM. *p<0.05, and **p<0.01.

Figure S7. Administration of the ketogenic diet restores insulin sensitivity and *miR-184* expression, related to Figure 7. (A) Blood glucose levels on 10-week old mice on chow (Chow) and ketogenic diet (Keto) (n=4-5). (B) Body weight quantification of 10-week old C57BL6 mice on chow and ketogenic diet (n=4-5). (C) β-cell mass analysis of 10-week old wild-type mice on chow (Chow) and ketogenic diet (Keto) (n=3-4). (D) Calculation of HOMA-IR and QUICKI indices for 10-week old *ob/ob* mice on chow (Chow/ob) and ketogenic diet (Keto/ob) (n=4-5). (E) Analysis of change in body weight of *ob/ob* mice on chow (Chow/ob) and ketogenic diet (Keto/ob) (n=4-6). (F) Ratio of Ki-67+ and insulin+ cells in *ob/ob* mice on chow (Chow/ob) and ketogenic diet (Keto/ob) (n=3). (G) Western blotting analysis of Cadm1 in isolated islets from *ob/ob* mice on chow (Chow/ob) and ketogenic diet (Keto/ob). Results presented as mean ± SEM. *p<0.05, **p<0.01, and ***p<0.001.

Supplemental Experimental Procedures

Computational Analysis.
Raw data from the Illumina scanner were loaded into R using the lumi package. Mappings to gene names and gene IDs was provided by the lumiMouseIDMapping package. Light intensities were quantile-normalized using the lumiN function of the lumi package. The analysis focused on probes for which the detection p-value smaller than 0.05 either in the TC control or at any of the time-points of the experiment. Subsequent analyses focused on these probes and the remaining was discarded. Mappings of probes to gene IDs were obtained from the lumiMouseAll.db package. Finally, the differential regulation in gene expression was calculated as the log2 fold change in signal intensity at the different time-points compared to the TC control. For investigating the effect of *miR-184* induction target genes, all genes were collected that carried a canonical *miR-184* binding site in the 3' UTR, defined as a heptamer complementary to positions 2-8 of the miRNA, or to positions 2-7 with a 'U' at position 1 (Bartel, 2009). 3' UTR sequences were downloaded from the RefSeq database (NCBI) on January 18, 2011. Among the genes collected “with seed” were the 50 genes carrying binding sites with highest probability of being under evolutionary selective pressure, as inferred by to ElMMo algorithm (ElMMo top 50) (Gaidatzis et al., 2007). In addition, a third group of genes was defined “without seed”, that did not carry a heptameric seed match to *miR-184*. For each of these groups of genes, the probability density function of log2 fold change was plotted as well as the 95% confidence interval on the mean log2 fold change. Finally, the tendency of genes “with seed” or in the “ElMMo top 50” was tested to be repressed compared to genes “without seed” using Wilcoxon's rank sum test and reported the resulting p-values in the figure legend. The NCBI Gene expression Omnibus (GEO) accession number which links to referenced Illumina array data is GSE46623.

Transmission electron microscopy
The whole pancreases were dissected, fixated in 4% paraformaldehyde and sliced with a scalpel to smaller sections (1–2 mm) as described previously (Wendt et al., 2012). Samples were incubated in 2.5% glutaraldehyde overnight and treated with 1% osmium tetroxide for 2 h, dehydrated, and then embedded in AGAR 100 (Oxford Instruments Nordiska AB, Sweden). Finally they were cut in 70- to 90-nm sections, put on Cu-grids, and contrasted with uranyl acetate and lead citrate. The samples were examined in a JEM 1230 electron microscope (JEOL-USA. Inc., Peabody, MA), and the micrographs were analyzed with respect to LDCV density and docked LDCV density as previously described (Vikman et al., 2009). The diameter of individual vesicles was determined using Scion Image (NIH freeware). The granule volume density (Nv) and surface density (Ns) were calculated using in-house software programmed in MatLab (version 7x).

Analytic Procedures.
Indirect calorimetry and locomotor activity was measured with the PhenoMaster System (TSE, Germany). Ex vivo islet secretion studies were performed with an automated perifusion system and measured by RIA (Biorep, USA).

Supplemental References
Bartel, D.P. (2009). MicroRNAs: target recognition and regulatory functions. Cell *136*, 215–233.

Gaidatzis, D., van Nimwegen, E., Hausser, J., and Zavolan, M. (2007). Inference of miRNA targets using evolutionary conservation and pathway analysis. BMC Bioinformatics *8*, 69.

Vikman, J., Jimenez-Feltström, J., Nyman, P., Thelin, J., and Eliasson, L. (2009). Insulin secretion is highly sensitive to desorption of plasma membrane cholesterol. FASEB J. *23*, 58–67.

Wendt, A., Speidel, D., Danielsson, A., Esguerra, J.L.S., Bogen, I.L., Walaas, S.I., Salehi, A., and Eliasson, L. (2012). Synapsins I and II are not required for insulin secretion from mouse pancreatic β-cells. Endocrinology *153*, 2112–2119.

Table S1. Small RNA sequencing profile of pancreatic islets from *ob/ob* mice and C57BL/6J littermates, **related to Figure 1** (Attached as an Excel File).

Table S2. Characteristics of human islet donors**, related to Figure 1.**

Sample ID	Sex	Age (years)	BMI (kg/m2)	Premortem diagnosis of T2D	Experiments
**HI 100**	Male	52	44	Yes	Fig.1I and Fig.1M qPCR
**HI 101**	Female	34	31	Yes	Fig.1I and Fig.1M qPCR
**HI 102**	Female	57	28	Yes	Fig.1I and Fig.1M qPCR
**HI 103**	Male	39	31	No	Fig.1I and Fig.1M qPCR
**HI 104**	Female	46	33	No	Fig.1I and Fig.1M qPCR
**HI 105**	Male	56	23	No	Fig.1I and Fig.1M qPCR
**HI 106**	Male	38	34	No	Fig.1I and Fig.1M qPCR
**HI 107**	Male	36	36	No	Fig.1I and Fig.1M qPCR
**HI 108**	Female	57	29	No	Fig.1I and Fig.1M qPCR
**HI 109**	Female	59	23	No	Fig.1I and Fig.1M qPCR
**HI 110**	Male	48	23	No	Fig.1I and Fig.1M qPCR
**HI 111**	Female	57	23	Yes	Fig.1I and Fig.1M qPCR
**HI 112**	Female	56	38.8	Yes	Fig.1I and Fig.1M qPCR
**HI 113**	Female	54	52.7	Yes	Fig.1I and Fig.1M qPCR
**HI 114**	Female	48	24.5	Yes	Fig.1I and Fig.1M qPCR
**HI 115**	Female	67	23.4	Yes	Fig.1I and Fig.1M qPCR
**HI 116**	Female	59	23.8	Yes	Fig.1I and Fig.1M qPCR
**HI 117**	Male	59	26.5	Yes	Fig.1I and Fig.1M qPCR
**HI 118**	Female	58	20.1	No	Fig.1I and Fig.1M qPCR
**HI 119**	Female	55	32.6	No	Fig.1I and Fig.1M qPCR
**HI 120**	Female	37	34.4	No	Fig.1I and Fig.1M qPCR
**HI 121**	Male	59	26.7	No	Fig.1I and Fig.1M qPCR
**HI 122**	Female	35	31.6	No	Fig.1I and Fig.1M qPCR
**HI 123**	Female	39	41.5	No	Fig.1I and Fig.1M qPCR
**HI 136**	Male	51	31	Yes	Fig.1I and Fig.1M qPCR
**HI 137**	Female	57	23	No	Fig.1I and Fig.1M qPCR
**HI138**	Male	51	31	No	Fig.1I and Fig.1M qPCR
**HI139**	Male	42	30.7	Yes	Fig.1I and Fig.1M qPCR
**HI140**	Female	41	35.5	No	Fig.1I and Fig.1M qPCR
**HI141**	Female	59	22.7	No	Fig.1I and Fig.1M qPCR
**HI142**	Female	27	21.1	No	Fig.1I and Fig.1M qPCR
